# Effect of salbutamol on neuromuscular junction function and structure in a mouse model of DOK7 congenital myasthenia

**DOI:** 10.1093/hmg/ddaa116

**Published:** 2020-06-16

**Authors:** Richard G Webster, An E Vanhaesebrouck, Susan E Maxwell, Judith A Cossins, Weiwei Liu, Ryo Ueta, Yuji Yamanashi, David M W Beeson

**Affiliations:** 1 Neurosciences Group, Nuffield Department of Clinical Neurosciences, Weatherall Institute of Molecular Medicine, John Radcliffe Hospital, University of Oxford, Oxford OX3 9DS, UK; 2 Division of Genetics, Department of Cancer Biology, Institute of Medical Science, The University of Tokyo, Tokyo 135-8550, Japan

## Abstract

Congenital myasthenic syndromes (CMS) are characterized by fatigable muscle weakness resulting from impaired neuromuscular transmission. β2-adrenergic agonists are an effective treatment for DOK7-CMS. DOK7 is a component within the AGRN-LRP4-MUSK-DOK7 signalling pathway that is key for the formation and maintenance of the synaptic structure of the neuromuscular junction (NMJ). The precise mechanism of action of β2-adrenergic agonists at the NMJ is not fully understood. In this study, we investigated whether β2-adrenergic agonists improve both neurotransmission and structural integrity of the NMJ in a mouse model of DOK7-CMS. *Ex-vivo* electrophysiological techniques and microscopy of the NMJ were used to study the effect of salbutamol, a β2-adrenergic agonist, on synaptic structure and function. DOK7-CMS model mice displayed a severe phenotype with reduced weight gain and perinatal lethality. Salbutamol treatment improved weight gain and survival in DOK7 myasthenic mice. Model animals had fewer active NMJs, detectable by endplate recordings, compared with age-matched wild-type littermates. Salbutamol treatment increased the number of detectable NMJs during endplate recording. Correspondingly, model mice had fewer acetylcholine receptor-stained NMJs detected by fluorescent labelling, but following salbutamol treatment an increased number were detectable. The data demonstrate that salbutamol can prolong survival and increase NMJ number in a severe model of DOK7-CMS.

## Introduction

Congenital myasthenic syndromes (CMS) encompass a group of genetic disorders that impair neuromuscular transmission and result in fatigable muscle weakness. More than 30 genes have now been found to underlie CMS (1). Many subtypes of CMS benefit from acetylcholinesterase inhibitors (2,3). Acetylcholinesterase inhibitors improve neuromuscular junction (NMJ) function by increasing the concentration of acetylcholine and the period of time that acetylcholine remains within the synaptic cleft. However, some CMS patients do not respond to acetylcholinesterase inhibition and administration of these inhibitors may even result in worsening. These CMS typically either harbour mutations in genes involved in assembly of post-synaptic structure, typically *DOK7*, (or rarely *AGRN*, *LRP4* or *MUSK*), or have gene mutations that are exacerbated by excess of acetylcholine, such as *COLQ* mutations and slow channel syndromes (2,4). Many of these CMS subtypes respond well to adrenergic agonists, although a positive response in the slow channel syndrome is rare, and the response for AGRN-CMS is modest (4–12). DOK7-CMS has a particularly marked response to β2-adrenergic agonists (4,8,9). Typically, ephedrine or salbutamol is the β2-adrenergic agonists of choice used in clinic. In DOK7-CMS, improvement of the myasthenic symptoms is gradual and often occurring over weeks to many months (8,9,13–15), which contrasts with the rapid mode of action of pyridostigmine or 3,4-diaminopyridine used frequently in other myasthenic conditions.

**Figure 1:**
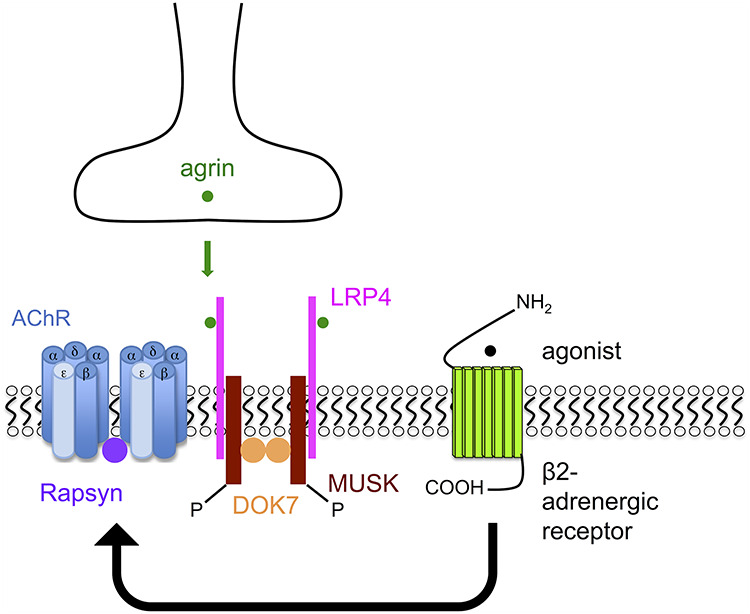
Postulated targets of action of β2-adrenergic agonists in DOK7 patients. DOK7 is part of the AChR clustering pathway. Simplified, DOK7 forms a dimer that interacts with and activates MUSK, and induces rapsyn-dependent AChR clustering, in cooperation with nerve-derived agrin (AGRN). This pathway is crucial for post-synaptic assembly. In DOK7 patients, the AChR clustering pathway and thereby post-synaptic assembly is impaired. The marked response of DOK7 patients to β2-adrenergic agonists suggests that β2-adrenergic agonists can compensate in some way for the impaired AChR clustering pathway (due to loss of DOK7 function). Thus, we hypothesize that β2-adrenergic agonists improve neurotransmission by stabilizing post-synaptic NMJ structure in DOK7 patients.

The mechanism through which salbutamol or other β2-adrenergic agonists improve neuromuscular transmission in patients with CMS is not well understood. It is plausible that β2-adrenergic agonists can partially compensate for disrupted post-synaptic structure, since the CMS subtypes that show the most marked response have mutations in genes encoding proteins that govern the formation and stability of the NMJ via the AGRN-induced LRP4-MUSK-DOK7 signalling pathway (Fig. 1). MUSK and its activator DOK7 are essential for the initial aggregation of pre-patterned acetylcholine receptors (AChRs) prior to nerve arrival, which is then maintained along with the complex post-synaptic structure through the AGRN-induced pathway (16,17). The mechanism through which β2-adrenergic agonists improve neurotransmission in DOK7-CMS may be through an effect on this pathway, or through independent stabilization of the synaptic structures.

The most prevalent DOK7-CMS mutation is the frameshift duplication c.1124_1127dupTGCC (p.Ala378Serfs) in exon 7 (18–21). It causes truncation of the C-terminal of DOK7, resulting in reduced phosphorylation and activation of MUSK (the key orchestrator of the AChR clustering pathway) and thus a disrupted post-synaptic structure (22,23). Here, we investigate the effect of β2-adrenergic agonists on NMJ function and structure in a mouse model homozygous for this mutation (24), characterizing the electrophysiological and morphological parameters in the response to salbutamol medication.

## Results

### Characterization of the DOK7-CMS model mice

**Figure 2:**
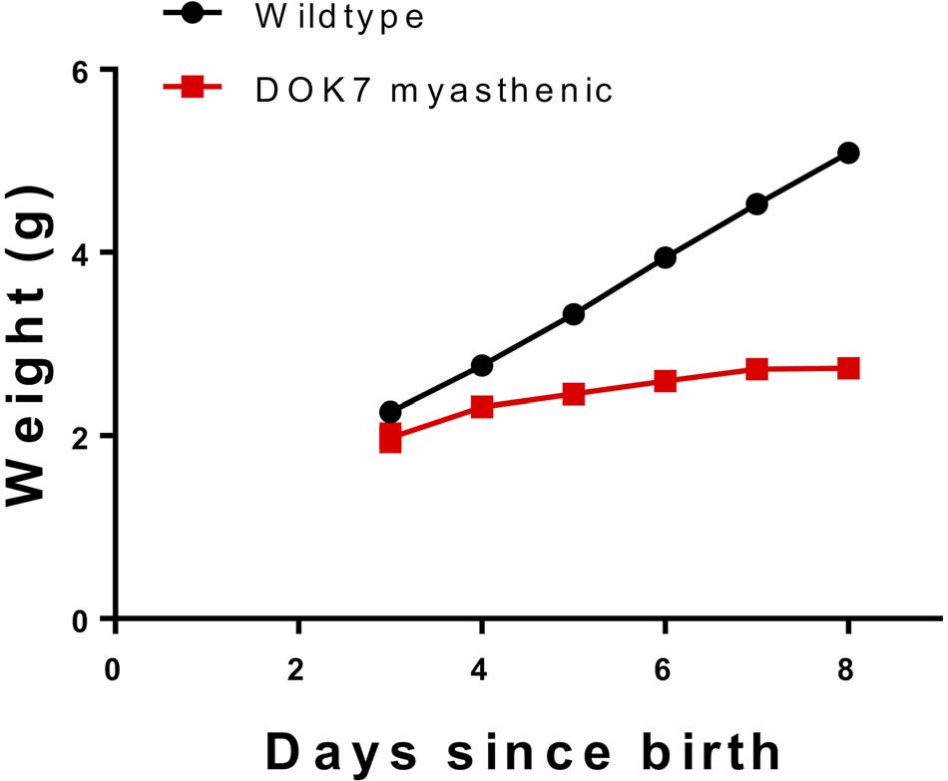
DOK7 myasthenic mice fail to gain weight at the rate of wild-type littermates following birth. Mean ± SEM, values for SEM are smaller than symbol size and are therefore obscured. Wild-type mice *n* = 109, DOK7 myasthenic mice *n* = 10.

Although CMS patients homozygous for c.1124_1127dupTGCC generally have a relatively mild phenotype, that is not the case for the mouse model harbouring the equivalent homozygous mutation, which is characterized by severe muscle weakness and a reported premature death around the age of postnatal day (P)13-P20 (24). First, we compared body weight of DOK7 myasthenic mice with wild-type mice from P3 to P8 or until humane endpoint. Humane endpoint was defined as: either (1) a weight loss of 0.1 g or more, or (2) no weight gain over 48 h. Body weight in DOK7-CMS model mice was significantly reduced from P4 compared with wild-type mice, and became more pronounced with each day thereafter (Fig. 2). Where body weight of DOK7-CMS model mice was 82% of wild-type values at P4, body weight at P8 was only 53% of wild-type values. At P8, DOK7-CMS model mice weight by average 2.7 g (±0.11), whilst wild-type mice weighed on average 5.1 g (±0.06). From this cohort, three model mice were culled before P8, because they reached the humane endpoint criteria of weight loss; their data were not included in the model mice average.

Neuromuscular transmission was assessed by electrophysiological analysis of hemidiaphragm–phrenic nerve preparations at P8. In contrast to wild-type mice, muscle contractions and muscle fibre action potentials following nerve stimulation could not be blocked by μ-conotoxin GIIIB (a selective Nav1.4 inhibitor (25)) in DOK7-CMS model mice. Consequently, only spontaneous miniature endplate potentials (mEPPs) could be recorded in DOK7 mice. mEPPs are small depolarizations of the post-synaptic membrane caused by the spontaneous release of a single synaptic vesicle containing acetylcholine. The incidence and frequency of mEPPs was monitored for 5 min for each impalement. Evoked endplate potentials (EPPs) are larger depolarizations of the post-synaptic membrane caused by the release of numerous synaptic vesicles following nerve stimulation. Isolated EPPs could not be recorded in DOK7 mice, because of the persistence of muscle contractions resulting in movement artefact and because of the preservation of the concurrent action potential obscuring the EPP despite μ-conotoxin GIIIB.

**Figure 3:**
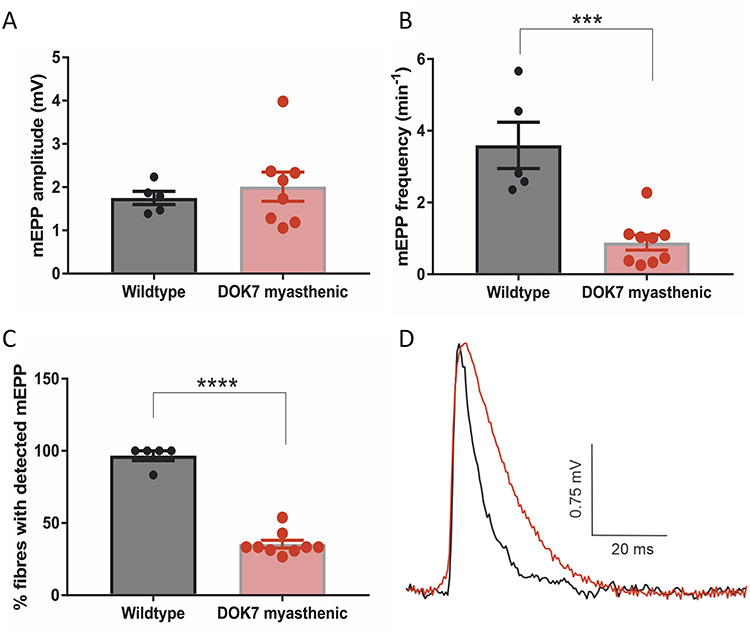
Comparison of diaphragm neurotransmission parameters in wild-type (*n* = 5) and DOK7 myasthenic mice (*n* = 9). (**A)** mEPP amplitude was not significantly different between wild-type and DOK7 myasthenic mice. However, mEPP frequency (among fibres with detectable mEPPs) and % of fibres with detectable mEPP (**B** and **C**) were significantly reduced in DOK7 myasthenic mice. Each data point represents the mean data from an individual mouse. ^***^P < 0.001, ^****^P < 0.0001. (**D)** Shows example of wild type (black) and DOK7 myasthenic (red) mEPP recordings, DOK7 myasthenic mEPP recording has a slower rate of decay.

We compared mEPP characteristics between DOK7-CMS model and wild-type mice (Fig. 3). Mean mEPP amplitude (derived from averaging all mEPP events per endplate) in DOK7 myasthenic mice (2.01 mV ± 0.34) was similar to wild-type mice (1.75 mV ± 0.15). Mean mEPP frequency was severely reduced in DOK7 myasthenic mice (0.88 per min ± 0.21), compared with wild-type mice (3.59 per min ± 0.65) (*P* < 0.001). mEPPs could be detected in fewer fibres in DOK7-CMS model mice (35.4% ± 2.7) than in wild-type mice (96.7% ± 3.3) (*P* < 0.0001). As illustrated by Figure 3D, decay times of mEPPs were significantly longer in DOK7-CMS model mice (14.9 ms ± 1.25) than in wild-type mice (7.16 ms ± 0.7) (*P* = 0.001) and correspondingly, decay rates were slower in DOK7 mice (−0.0.05 mV/ms ± 0.066) than in wild-type mice (−0.08 mV/ms ± 0.012) (*P* = 0.02). This suggests the persistent presence of the γ-subunit of the AChR in DOK7-CMS model mice (26). In wild-type mice, in contrast, many fetal AChR containing the γ subunit would have been replaced by AChR harbouring the adult ε-AChR subunit—characterized by faster decay times—by the time of recording (P8), as the main switch is thought to occur between P5 and P7 in mouse diaphragm muscles (27).

**Figure 4:**
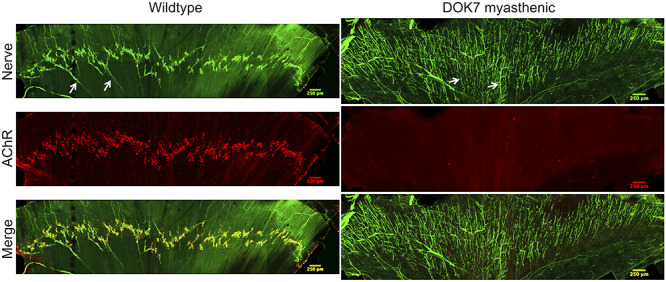
Wholemount staining of the endplate region illustrates the NMJ pattern and terminal nerve distribution in hemidiaphragms of wild-type and (untreated) DOK7 myasthenic mice at P8 (stitched tiles, scale bar = 250 μm). The left hemidiaphragms are shown. Neurofilament and synaptophysin antibodies were used to stain axon fibres and nerve terminals, respectively (green), visualized with Alexa Fluor 488 antibodies. Tertiary nerve terminals (which are aberrant in DOK7 mice) are indicated with arrows. α-Bungarotoxin conjugated to Alexa Fluor 594 was used to stain AChRs (red).

**Figure 5:**
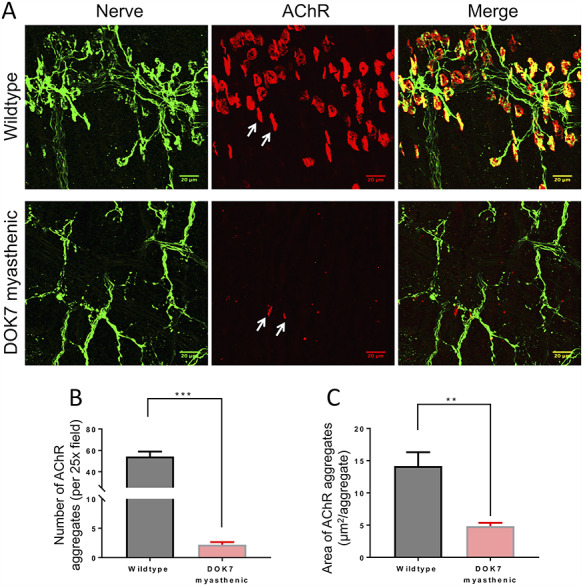
Fluorescent labelling of diaphragm NMJs in wild-type and (untreated) DOK7 myasthenic mice at P8 at higher magnification (scale bar = 20 μm). (**A)** Neurofilament and synaptophysin antibodies were used to stain axon fibres and nerve terminals, respectively (green). α-Bungarotoxin was used to stain AChRs (red). Some AChR plaques are indicated with arrows. Bar graphs show the number of AChR aggregates per field (**B**) and AChR-stained area per NMJ (**C**). ^**^P < 0.01, ^***^P < 0.001. Wild-type mice *n* = 13, DOK7 myasthenic mice *n* = 7.

At P8, whole-mount diaphragm muscles were compared between wild-type and DOK7-CMS model mice (Figs 4 and 5). Neurofilament and synaptophysin antibodies were used to stain axon fibres and nerve terminals, respectively. Post-synaptic AChRs were visualized by staining with α-bungarotoxin. Phrenic nerve distribution on tiled scans of hemidiaphragms was visually compared, and area and number of AChR aggregates per field were counted on individual images along the endplate region. In wild-type and DOK7 mice, the primary branch of the phrenic nerve entered the hemidiaphragm muscle and divided into two main branches, called secondary branches. In diaphragm muscles of wild-type mice, pretzel-shaped AChR aggregates and their nerve terminals (or tertiary nerve branches) were aligned in a narrow band along the two main (secondary) phrenic nerve branches, i.e. approximately in the middle of muscle fibres (Fig. 4). The innervation pattern of DOK7-CMS model mice was disorganized: tertiary nerve terminals were aberrant with long nerve terminals reaching to the costal edges of the diaphragm muscle (Fig. 4) and small nerve boutons that often did not form full contact with post-synaptic AChRs (Supplementary Material, Fig. S1). In addition, plaque-like AChR aggregates were no longer localized within a narrow band along the phrenic nerves, as in wild-type mice, but were widely spread between the middle and the costal edges of the hemidiaphragm.

More detailed example images from DOK7-CMS model and wild-type mice are shown in Figure 5A. Figure 5B demonstrates an obvious decrease in the number of AChR-stained NMJs per field in DOK7-CMS model mice (2.2 ± 0.44), compared with wild-type mice (54.30 ± 4.60) (*P* < 0.001). Only a few AChR aggregates per field could be imaged in the DOK7-CMS model mice. As illustrated by Figure 5C, AChR aggregates were smaller (4.85 μm^2^ ± 0.51) than in wild-type mice (14.18 μm^2^ ± 2.14) (*P* < 0.01). Most AChR aggregates were in close proximity of a nerve terminal bouton, and therefore can be considered as the post-synaptic portion of the NMJ.

The phenotype of the DOK7-CMS model mouse is markedly more severe than the phenotype of humans carrying the *DOK7* 1124_1127dupTGCC mutation. The unexpected severity of this phenotype places limitations on the utility of this model. However, we investigated the efficacy of salbutamol to alleviate the lethality and disruption of neurotransmission in this model.

### Salbutamol enhances neurotransmission and NMJ abundance in DOK7-CMS model mice

Salbutamol was dissolved in saline (40 μL) and administered via daily intraperitoneal injection. We based our salbutamol dose range on a previous study, in which a dose of 8 mg/kg was shown to be effective in increasing strength in a MUSK myasthenia gravis mouse model (28). In the initial phase of this study, increasing doses of salbutamol were used, i.e. 4, 8 and 16 mg/kg/day. A saline-injected group was used as control group. DOK7-CMS model mice were randomly assigned to the four groups: (1) saline, (2) 4 mg/kg salbutamol, (3) 8 mg/kg salbutamol or (4) 16 mg/kg salbutamol.

Mice were injected once daily from P4 to P7. They were weighed daily from P3 to P8 or until humane endpoint (a weight loss of 0.1 g or more, or no weight gain over 48 h). DOK7 mice were too weak for evaluation of muscle strength. At P8, mice were euthanized, and diaphragm muscles were collected for endplate potential recording and evaluation of NMJ morphology by fluorescent staining.

**Figure 6:**
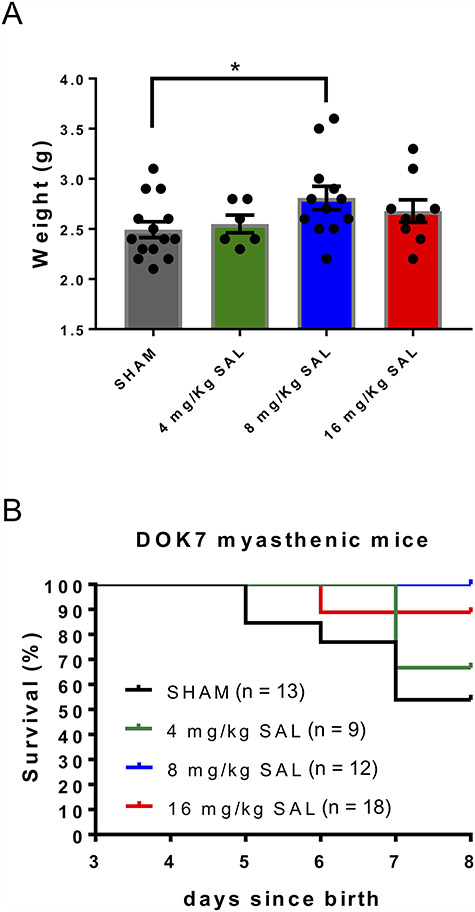
Salbutamol improves weight gain and survival (time until end of trial at P8 or until humane endpoint). DOK7 myasthenic mice were injected with increasing doses of salbutamol (SAL) or saline (SHAM). Body weight was monitored daily. (**A**) Salbutamol at 8 mg/kg significantly increased body weight in DOK7 mice compared with SHAM at P8. ^*^P < 0.05. (**B)** In addition, salbutamol significantly increased survival. *P* < 0.01 for 8 mg/kg and *P* < 0.05 for 16 mg/kg.

We investigated whether salbutamol treatment affected body weight in DOK7-CMS model mice (Fig. 6A). Salbutamol significantly increased body weight at 8 mg/kg (*P* < 0.05), but not at 4 or 16 mg/kg. At P8, body weight of DOK7 myasthenic mice injected with 8 mg/kg salbutamol was 2.8 g (±0.12), compared with 2.5 g (±0.08) in saline injected DOK7-CMS model mice.

During the trial, some control (untreated) animals were culled prior to P8 as they reached humane endpoint criteria. Since these weaker/smaller untreated mice were excluded from the completed analysis, mean weight would have underestimated the beneficial effect of drug treatment. Therefore, we analyzed time until humane endpoint, in which an animal was euthanized because of weight loss of more than 0.1 g or absence of weight gain for more than 48 h. Time to humane endpoint was used as an estimate of survival time.

We observed that 46% of saline-injected mice reached humane endpoints before P8, with humane endpoints reached between P5 and P7 (Fig. 6B). Stressful stimuli during the neonatal period, such as saline injection, likely contributed to a higher number of saline-injected DOK7-CMS model mice reaching humane endpoints compared with untreated DOK7-CMS model mice previously analyzed (33% loss). A significant improvement in time to humane endpoint was observed in salbutamol-injected mice at doses of 8 (*P* < 0.01) and 16 mg/kg (*P* < 0.05), but not at 4 mg/kg, compared with saline-injected mice. Thus, salbutamol significantly improved the survival of DOK7 myasthenic mice in this initial trial.

### Salbutamol increases the number of muscle fibres with detectable mEPPs in the diaphragm of DOK7-CMS model mice

**Figure 7:**
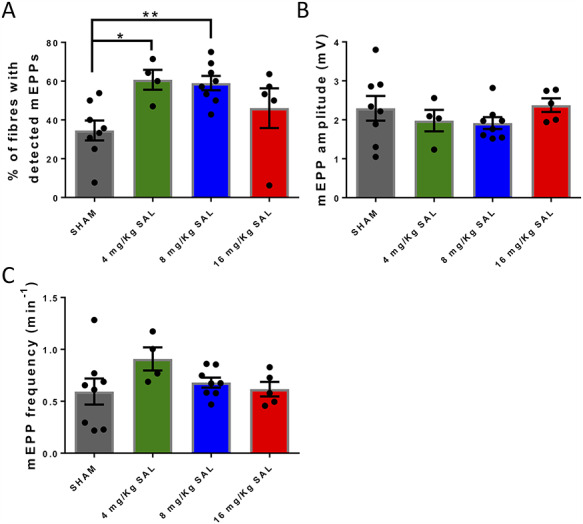
Salbutamol improves some parameters of neurotransmission in diaphragm muscle. (**A**) Shows that salbutamol increases the percentage of fibres with detectable mEPPs (4 and 8 mg/kg), but did not alter mEPP amplitude or mEPP frequency (**B** and **C**). ^*^P < 0.05, ^**^P < 0.01.

mEPP amplitude, mEPP frequency and the proportion of fibres with detectable mEPPs were compared between salbutamol- and saline-injected mice (Fig. 7). Salbutamol treatment significantly increased the number of fibres with detectable mEPPs, at doses of 4 and 8 mg/kg (Fig. 7A). The mEPPs could be detected in 60.7% in mice injected with 4 mg/kg salbutamol and in 59.0% in mice injected with 8 mg/kg salbutamol, versus 34.5% in mice injected with saline. However, salbutamol treatment did not alter mEPP amplitude or mEPP frequency (Fig. 7B and C).

### Salbutamol increases the number of AChR-stained NMJs detected by α-bungarotoxin staining in the diaphragm of DOK7-CMS model mice

**Figure 8:**
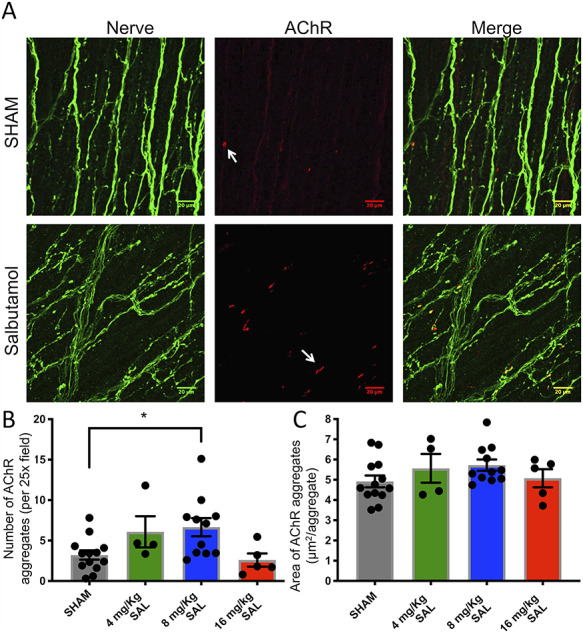
Salbutamol at 8 mg/kg significantly increased the number of NMJs observed in diaphragm muscle. (**A**) Fluorescent staining of NMJs of whole-mount diaphragms in DOK7 myasthenic mice, sampled at P8. Mice were injected daily from P4 until P8 with salbutamol (8 mg/kg) or saline (SHAM). Neurofilament and synaptophysin antibodies were used to stain axon fibres and nerve terminals, respectively (green). Post-synaptic AChRs of NMJs were stained with α-bungarotoxin (red). Some AChR plaques are indicated with arrows. Scale bar = 20 μm. Bar graphs show number of AChR aggregates per field (**B**) and AChR-stained area per NMJ (**C**). 8 mg/kg salbutamol significantly increased the number of AChR aggregates compared with SHAM-injected mice. ^*^P < 0.05.

We investigated whether the increase in weight and time to humane endpoint observed with salbutamol treatment related to changes in NMJ morphology. Innervation was disordered in all groups (saline and salbutamol) with nerve terminal distribution widespread (Fig. 8A, Supplementary Material, Fig. S2) and tertiary nerve terminals reaching the costal edges of the diaphragm muscle (as in Fig. 4). Figure 8B demonstrates a significantly increased number of AChR aggregates per field in salbutamol-injected mice, at a dose of 8 mg/kg (6.65 ± 1.12), but not at 4 or 16 mg/kg, compared with saline-injected mice (3.22 ± 0.58) (*P* < 0.05). No significant difference in the AChR-stained areas was found between salbutamol- and saline-injected groups (Fig. 8C). Most AChR aggregates (96% by average) were in proximity of a nerve terminal, without differences between groups.

### Salbutamol prolongs survival of DOK7-CMS model mice

**Figure 9:**
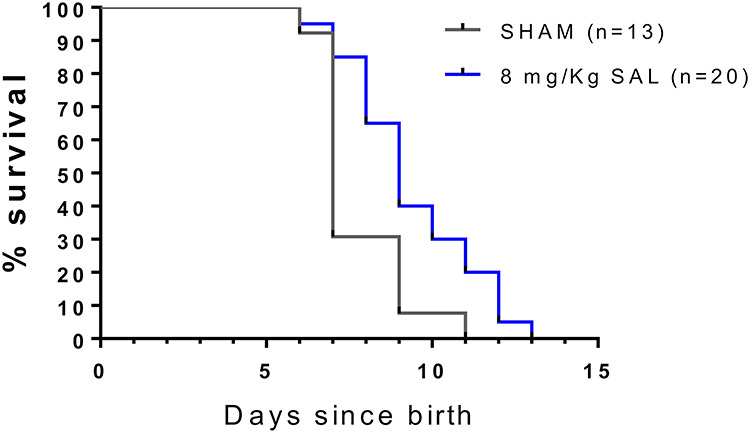
Salbutamol treatment daily from P4 improved DOK7 myasthenic mice survival (*P* = 0.01). Salbutamol (8 mg/kg) or SHAM injection was administered daily from P4. Survival was recorded as number of days since birth until defined humane endpoint criteria were met.

Since 8 mg/kg salbutamol was consistently the most effective dose, we determined if this dose of salbutamol could prolong the lifespan of DOK7-CMS model mice beyond the previous fixed endpoint of 8 days. Model mice were injected daily from P4 with either 8 mg/kg salbutamol or saline control and monitored until the criteria for humane endpoint were reached. Figure 9 shows survival curves for these groups of mice; 8 mg/kg salbutamol significantly prolonged the time to necessary humane endpoint (*P* = 0.01). Mean survival for salbutamol treated mice was increased to 9 days compared with 7 days when injected with saline alone. There was no difference in survival time in response to treatment between male and female mice. Mean survival time for salbutamol treatment group was 9.71 and 9.54 days for male and female myasthenic mice, respectively; whilst in the saline group it was 7.33 and 8.11 days for male and female myasthenic mice, respectively.

## Discussion

We show in a vertebrate model of DOK7-CMS that the β2-adrenergic agonist salbutamol improves neurotransmission and neuromuscular structure. An increase in the number of detectable NMJs following treatment suggests there is enhanced stability of the synaptic structure.

The effect of *DOK7* mutations on AChR clustering and assembly of post-synaptic structure has been well studied in muscle cell cultures and mice (18,19,24,29). DOK7 also plays a role in pre-patterning (formation of endplate band in middle of muscle fibre) and axon pathfinding during development (29). All these features were impaired in the DOK7-CMS mouse model: NMJs were scarce and abnormally small, and NMJs were no longer localized in a narrow central band, and the span of tertiary nerve terminal arborization was enlarged. Alterations of post-synaptic structure may also alter retrograde signalling to the pre-synaptic nerve terminals, as indicated by the observation of small nerve terminals in DOK7-CMS model mice. This is characteristic of human DOK7 patients, where NMJs and nerve terminals on motor-point biopsies are abnormally small (4,19,21,30,31). Although DOK7-CMS model mice have a similar genotype to humans, the phenotype is more severe. The underlying cause of this difference is not known.

These are the first electrophysiological data generated from this mouse model. Few electrophysiological studies have been reported in human DOK7-CMS (19,21,30). The main electrophysiological features are reduced amplitude of EPP, resultant from reduced quantal content, but unchanged mEPP amplitude. Equally, in the DOK7-CMS model mouse, no reduction in mEPP amplitude was observed. This reflects that AChR density at the NMJ is relatively unaffected by the *DOK7* mutation, as previously shown in motor-point biopsies in DOK7 human patients by AChR radiolabeling (19). However, a reduction in mEPP frequency and in the number of active NMJs was found in the DOK7-CMS model mice. Reduced mEPP frequency correlates with the smaller NMJ size and thus fewer vesicle release sites. The reduced proportion of active NMJs (muscle fibres from which mEPPs can be recorded) correlates with the scarce number of NMJs found on immunohistochemistry. The effect of the *DOK7* mutation on mEPP frequency or the proportion of active NMJs has not been previously reported. EPPs could not be recorded in DOK7-CMS model mice, because action potentials could not be abolished by NaV1.4 blockade. We presume this is due to an altered developmental profile with immaturity of the NMJ, that likely still utilize the fetal form NaV1.5 (instead of the adult form NaV1.4) in the junctional folds to initiate the muscle action potential, which are insensitive to μ-conotoxin GIIIB blockade.

We found that salbutamol increases the number of active NMJs in the DOK7-CMS model. Electrophysiological changes following salbutamol correlated with an increase in the number of fluorescent labelled NMJs in diaphragm muscles of the model. Our findings are in accordance with two recent articles using DOK7 mutant and knockdown models, in which the authors showed that 24 h of salbutamol administration increased the number of AChR clusters in myotubes and zebrafish embryos, respectively (32,33). Other studies using other myasthenic models also showed increased post-synaptic areas, i.e. in a MuSK myasthenia gravis mouse model, a slow channel model and recently in an AChR-deficient and ColQ-deficient mouse model (28,34–36). Although we noted an increase in post-synaptic area on some diaphragm images in salbutamol-treated mice, overall this was not reflected in statistically significant differences. Improvement in electrophysiological and morphological features following salbutamol was reflected by an improvement in mouse weight gain and thereby prolonged time to humane endpoint in mice treated with salbutamol.

Administration of salbutamol did not result in overt changes of nerve arborization in diaphragm muscles in this study. This contrasts with a study performed in DOK7 knockdown zebrafish embryos, where the authors showed that salbutamol partially rescued impaired developmental nerve distribution (axon pathfinding) (32). In addition, in the latter study an increase in the area of co-localization between post-synaptic AChR area and its nerve terminal was observed following treatment with salbutamol. In other myasthenic mouse models, similar findings were observed (28,35). In the DOK7 mouse model used in this study, detailed nerve-AChR colocalization studies would be technically challenging and would require high-resolution microscopy, because of the small size of the endplates.

We cannot fully exclude an additional effect of salbutamol on the muscle itself that might have contributed to the increased survival and weight in DOK7 mice. Most previous studies, however, did not find a significant effect of salbutamol or related drugs on muscle fibre hypertrophy or fibre type switching in myasthenic models (35–37).

The overall effect at the highest dose of salbutamol was less effective than at 8 mg/kg. This might be due to the number of animals used or, more likely, due to the fact that this high dose resulted in full occupancy of β2-adrenergic receptors and thereby desensitization of the receptors (38). Only small differences in post-synaptic structure between salbutamol- and saline-injected groups were observed. It is likely that the duration of treatment attainable was too short, due to the severity of the mouse model used. As stated, peak effects of salbutamol in DOK7-CMS can take several months to fully develop (39). We show an increase in the number of detectable NMJs following salbutamol, indicating improvement in stability of the post-synaptic structure. It is very likely that the β2-adrenergic receptor also plays an important role in maintenance of the post-synaptic structure in physiological circumstances. This idea is further strengthened by the recent discovery of the presence of sympathetic innervation and β2-adrenergic receptors close to NMJs and their microcirculation (34,40,41). Moreover, therapeutic β-adrenergic receptor blocking drugs are known to be harmful for many myasthenic conditions.

Our results support the use of β2-adrenergic agonists in DOK7-CMS. We show that the β2-adrenergic agonist salbutamol can act to partially compensate for impairment within the AChR clustering pathway due to loss of DOK7 function. This study provides evidence that β2-adrenergic receptors affect components of the NMJ that play a role in synaptic maintenance, and thereby improves neurotransmission and clinical phenotype.

## Materials and Methods

### Ethical statement, mouse breeding and genotyping

All mouse experiments described were conducted at the University of Oxford under a project licence from the UK Home Office.

A DOK7-CMS mouse model, homozygous for the frameshift mutation c.1124_1127dupTGCC (p.Ala378Serfs) corresponding to the most prevalent mutation in patients, was used. Mice were generated as previously described (24). The *Dok7* mutant mouse gene was driven from its natural mouse promoter.

Heterozygous mice were mated to siblings of the same genotype to generate litters containing mice homozygous for c.1124_1127dupTGCC. Homozygous mutant mice are further called DOK7-CMS model mice. As the phenotype between homozygous wild-type mice and heterozygous mutant mice is indistinguishable, they are both referred to as wild-type mice.

Genotyping of mice was performed by PCR-restriction fragment length polymorphism analysis. Neonatal mice were tail-clipped and adult mice were ear-clipped. DNA of those tissues was extracted using Direct PCR Extraction Reagent (Viagen Biotech). The transgene specific region was then amplified by PCR, using AccuPrime GC-rich DNA polymerase. The primer pairs used were 5′ ATAGAGGCTGGCTTGGCAGATG 3′ and 5′ TCCTAGCCTAACCATTGTGACTAC 3′. PCR products were digested with the restriction enzyme BamHI. Digested and undigested products (as control) were run on an agarose gel by electrophoresis. As the *DOK7* mutation created a BamHI restriction enzyme site (which is not present in wild-type mice), analysis of the agarose gel was then used to identify genotype of mice.

Gender in neonatal mice was determined by PCR of gDNA, as previously described (42). In brief, X-chromosomal *Xlr* and Y-chromosomal *Sly* genes were amplified by PCR, using primer pairs 5′-GATGATTTGAGTGGAAATGTGAGGTA-3′ and 5′-CTTATGTTTATAGGCATGCACCATGTA-3′. PCR products were analyzed on 2% agarose gels.

### *Ex-vivo* electrophysiology of phrenic nerve-diaphragm preparations

Recording of phrenic nerve/hemi-diaphragm preparations were performed, as previously described (43). Phrenic nerve/hemi-diaphragm preparations were dissected and bathed in Kreb’s solution, bubbled with 95% O_2_/5% CO_2_. The composition of the Kreb’s solution was as follows: 118 mm NaCl, 4.7 mm KCl, 1.2 mm MgSO_4_.7H_2_O, 1.2 mm KH_2_PO_4_, 24.9 mm NaHCO_3_, 11.1 mm D-glucose and 2.5 mm CaCl_2_.

Usually, the right hemidiaphragm was used for recording. The phrenic nerve was pulled into a suction electrode, which was coupled to a pulse generator (GRASS instruments S48 square wave stimulator, Quincy, USA), with an associated stimulus isolation unit. Recording electrodes were connected to an Axoclamp 900A amplifier (Molecular Devices, California, USA). Data signals passed through a HumBug 50 Hz noise eliminator (Quest Scientific via Digitimer, Welwyn Garden City, UK). Recorded mEPPs and EPPs were continuously digitized at 10 kHz sampling rate and filtered at 4 kHz, using Axon Digidata 1322A interface (Molecular Devices, USA) controlled by pClamp 10 software (Molecular Devices, USA).

To block action potentials, μ-conotoxin GIIIB at 2.5 μM was added to the bath of each hemidiaphragm preparation for 30 min (Peptide Institute Inc., Japan). The excess unbound toxin was washed out before recordings started. Recordings were made at room temperature (RT, 22–23°C).

Depolarizations at the endplate were recorded intracellularly using a single sharp electrode. Electrodes were borosilicate glass micropipette electrodes (1.5 mm Outer Diameter, 1.17 mm Inner Diameter). Electrodes were pulled by a programmable P-97 microelectrode puller (Sutter Instruments, Novato, CA) and filled with 3 M KCl. Filled electrodes had a resistance of ~ 15 MΩ. Electrodes were positioned above endplate regions, as visualized by stereomicroscope (Olympus BX51WI) under micromanipulator control (Scientifica, UK). To evoke an EPP, the phrenic nerve was stimulated via two silver-wire electrodes.

If the membrane potential depolarized below −50 mV, the recording was abandoned for that endplate. Recordings or part of recordings with an unstable or drifting membrane potential were excluded from analysis. Recording time was up to 1 h30 per animal. At least 10 endplates per animal were sampled.

Offline analysis was later performed blinded to treatment group. Each mEPP was detected via template or threshold searching in Clampfit 10 software. All mEPP amplitude measurements were adjusted for deviation of a resting membrane potential of −80 mV, to correct for changes in driving force due to an altered post-synaptic membrane potential.

### Morphological analysis of NMJs by fluorescent staining

#### Fluorescence staining protocol

Diaphragm muscles were dissected in Kreb’s buffer, bubbled with 95% O_2_/5% CO_2_. The non-recorded hemidiaphragm was pinned out on blocks of Sylgard. Tissues were fixed for 30 min at RT in PBS containing 1% formaldehyde, immediately following dissection. Tissues were then briefly rinsed and kept in PBS at 4°C until further staining.

Samples were stained for AChR, neurofilament and synaptophysin, as previously described (37). Following each staining step, samples were thoroughly washed. In brief, muscles were incubated overnight at 4°C with α-bungarotoxin Alexa Fluor® 594 conjugate (Invitrogen, B13423, 1:150). Muscles were permeabilized and blocked by brief incubation in ice-cold methanol, followed by incubation in PBS-T (PBS with 0.05% Tween 20) containing 3% goat serum (Sigma) and 0.1% triton-X 100 for 30 min at RT. They were then incubated at 4°C overnight with chicken anti-neurofilament antibody (ab-4680, Thermo Fisher Scientific, 1:1000) and rabbit anti-synaptophysin antibody (ab-4, Thermo Scientific, 1:100). The next day, samples were incubated for 3 h at RT with goat anti-chicken and goat anti-rabbit Alexa Fluor® 488-conjugated secondary antibodies (Invitrogen, 1:100). Diaphragm muscles were whole-mounted.

All samples were mounted under fluorescence mounting media (Confocal Matrix, Micro-Tech-Lab). Slides were labelled with a code with no identifying information and randomized.

#### Imaging NMJs by confocal fluorescence microscopy

Images of NMJs were taken on an inverted confocal microscope, using a 25x oil objective (Zeiss LSM 880). Images were captured with Zen 2.3 LSM software. Identical image settings were used for all sections. Regions with nerve boutons from the tertiary nerve terminals were selected between the middle and the costal border of the hemidiaphragm. Z-stacks were collapsed to single images by maximum intensity projection. Pictures were taken by the investigators (AV and SM), unaware of genotype or treatment.

A stitched tile scan of the whole diaphragm was acquired overnight, using a 25x objective.

#### Morphological analysis of immunolabelled NMJs

Images were later quantitatively analyzed in a blinded and randomized fashion, using Fiji (44). For images of endplates obtained during experiments of DOK7 myasthenic mice, the following parameters were measured: the number of AChR aggregates per 25x field and their size. We made a macro with Fiji software for semi-automatization of the above measurements (44). Size filter was set as ‘1.75-infinity μm^2^’ and circularity filter as ‘0.0–0.6’.

### Statistical analysis

One-way or two-way ANOVA with Dunnett (using saline as reference group) or Sidak correction (no reference group) was used to compare the NMJ parameters between groups, with drug dose and/or time (days) as independent variables, as appropriate. If means of only two groups were compared, an unpaired (2-tailed) *t*-test was used. Values were presented as means and errors as SEMs, unless otherwise stated. Statistical significance was set at *P* < 0.05. For analysis of time to humane endpoint, Kaplan–Meier curves were computed, and a log-rank test was used to compare time with humane endpoint between groups.

## Supplementary Material

Figure_S1_ddaa116

Figure_S2_ddaa116
