# Supplementary figures and images for: Effect of salbutamol on neuromuscular junction function and structure in a mouse model of DOK7 congenital myasthenia
Source: Hum Mol Genet. 2020 Jun 16;29(14):2325–36. doi: 10.1093/hmg/ddaa116 (PMC7424765; doi:10.1093/hmg/ddaa116)

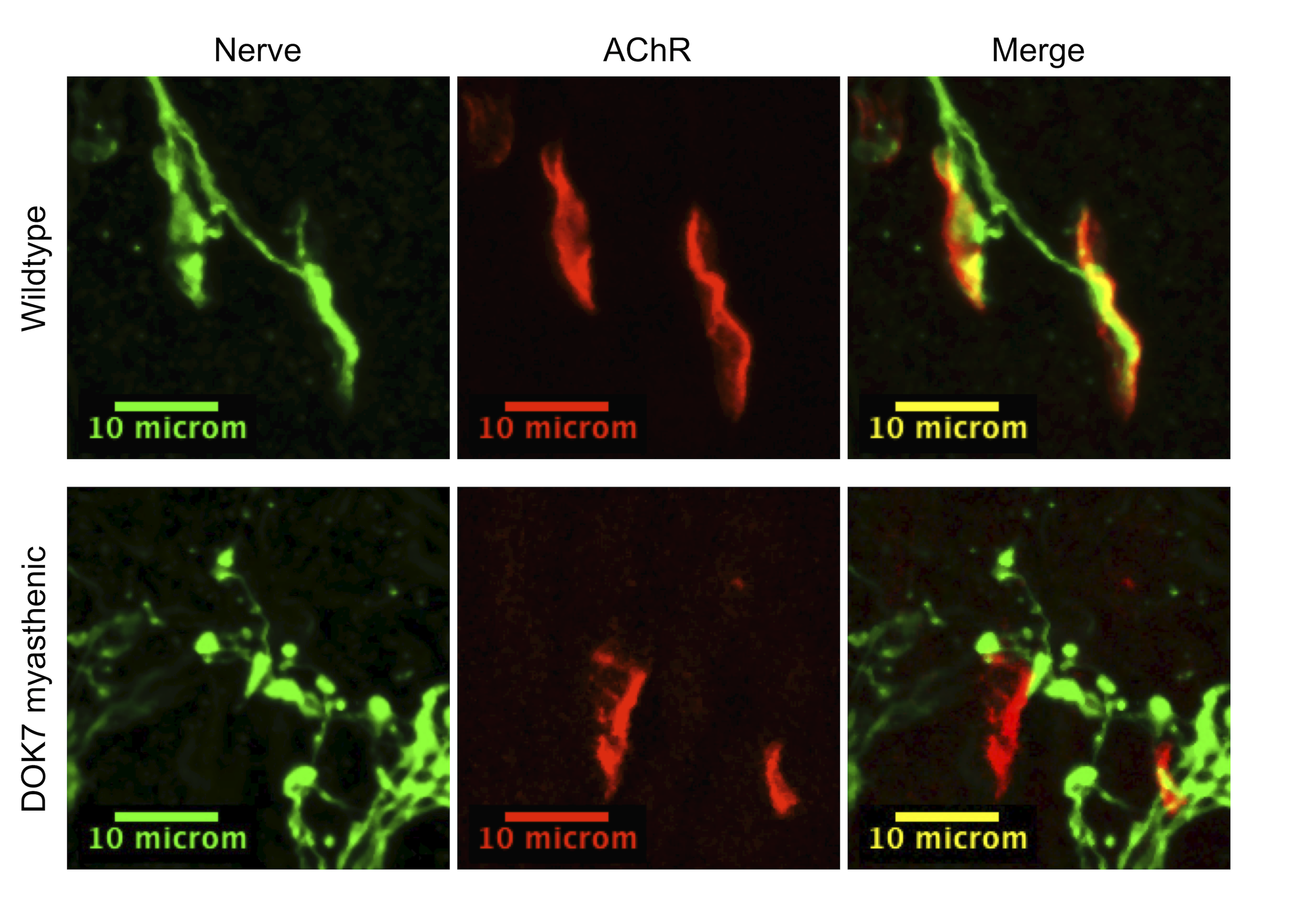

Supplement: Figure_S1_ddaa116 [file figure_s1_ddaa116.png]

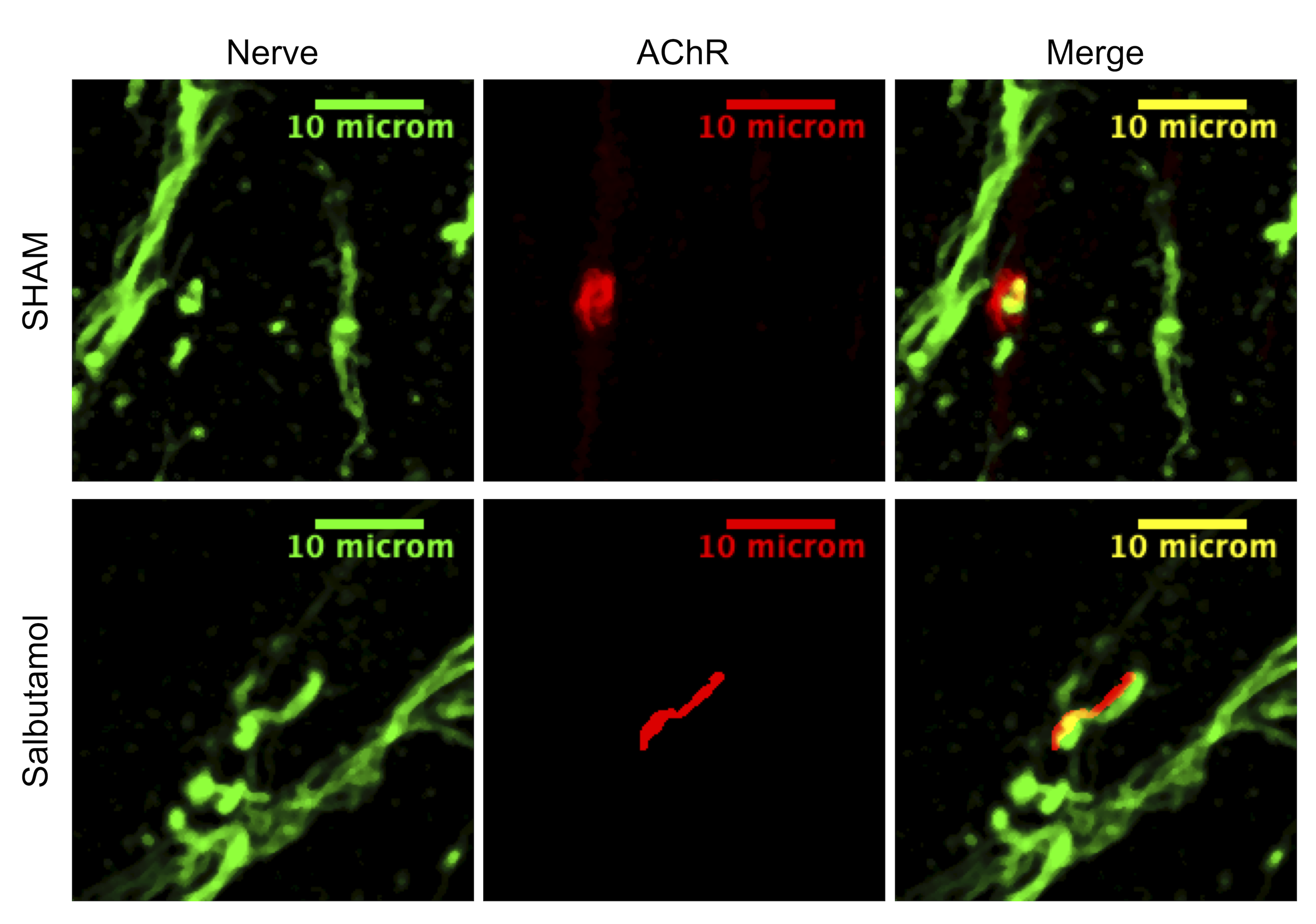

Supplement: Figure_S2_ddaa116 [file figure_s2_ddaa116.png]
